# Assessment of a newly developed immunochromatographic assay for NDM-type metallo-β-lactamase producing Gram-negative pathogens in Myanmar

**DOI:** 10.1186/s12879-019-4147-4

**Published:** 2019-06-28

**Authors:** Tatsuya Tada, Jun-ichiro Sekiguchi, Shin Watanabe, Kyoko Kuwahara-Arai, Naeko Mizutani, Izumi Yanagisawa, Tomomi Hishinuma, Khin Nyein Zan, San Mya, Htay Htay Tin, Teruo Kirikae

**Affiliations:** 1 Department of Microbiology, Juntendo University School of Medicine, 2-1-1 Hongo, Bunkyo-ku, Tokyo, 113-8421 Japan; 2 Microbiology Research Division, Kohjin Bio, Co., Ltd. Chiyoda, Saitama, Japan; 3 Department of Microbiome Research, Juntendo University Graduate School of Medicine, Tokyo, Japan; 4 National Health Laboratory, Yangon, Myanmar

**Keywords:** Immunochromatographic assay, Carbapenem-resistant gram-negative bacteria, NDM producers

## Abstract

**Background:**

To detect carbapenemase-producing Gram-negative bacteria in bacterial laboratories at medical settings, a new immunochromatographic assay for New Delhi metallo-β-lactamases (NDMs) was developed.

**Methods:**

The immunochromatographic assay for New Delhi metallo-β-lactamases producers was developed using rat monoclonal antibodies against NDMs. The assessment was performed using 350 isolates of Gram-negative bacteria, including *Acinetobacter baumannii* (51 isolates), *Enterobacteriaceae* (163 isolates), and *Pseudomonas aeruginosa* (136 isolates) obtained from 2015 to 2017 in medical settings in Myanmar. Of them, 302 isolates were resistant to carbapenems, including imipenem and/or meropenem. The *bla*_NDM_ genes were identified by PCR and sequencing.

**Results:**

Of the 350 clinical isolates tested, 164 (46.9%) (60 isolates of *Escherichia coli*, 51 isolates of *Klebsiella pneumoniae*, 25 isolates of *Enterobacter cloacae*, 23 isolates of *P. aeruginosa*, and 5 isolates of *A. baumannii*) were positive on this assay, and all the positive isolates harbored genes encoding NDM-1, − 4, − 5 and − 7. The remaining 186 (53.1%) isolates negative on the assay did not harbor genes encoding NDMs. The assay had a specificity of 100% and a sensitivity of 100%. The assessment revealed that more than 90% of carbapenem-resistant *Enterobacteriaceae* produced NDMs.

**Conclusions:**

The immunochromatographic assay is an easy-to-use and reliable kit for detection of NDMs-producing Gram-negative bacteria. The assay revealed that NDM-producing *Enterobacteriaceae* isolates are wide-spread in medical settings in Myanmar.

**Electronic supplementary material:**

The online version of this article (10.1186/s12879-019-4147-4) contains supplementary material, which is available to authorized users.

## Background

Metallo-β-lactamases (MBLs) are produced by many species of Gram-negative bacteria, as well as some species of Gram-positive bacteria, including *Bacillus* spp. [1, 2]. MBLs reduce susceptibility to carbapenems, cephalosporins, and penicillines except for monobactams [3]. New Delhi metallo-β-lactamase-1 (NDM-1) was initially detected from strains *Klebsiella pneumoniae* and *Escherichia coli* in 2008 in Sweden. Subsequently, NDM-1-producing *Enterobacteriaceae*, *Acinetobacter baumannii* and *Pseudomonas aeruginosa* were detected worldwide [4, 5]. Up to now, 21 NDM variants have been identified in Gram-negative pathogens in several countries (ftp://ftp.ncbi.nlm.nih.gov/pathogen/betalactamases/Allele.tab).

In the previous study, *Enterobacteriaceae*, including *Citrobacter freundii*, *Enterobacter* spp., *E. coli* and *K. pneumoniae*, producing NDM-1, NDM-4, NDM-5 or NDM-7 were isolated in medical settings in Myanmar [6–9]. To detect carbapenemase-producing Gram-negative bacteria in bacterial laboratories at medical settings in Myanmar, a new immunochromatographic assay for NDMs was developed and evaluated using Gram-negative bacteria in medical settings in Myanmar.

## Methods

### Bacterial strains

Strains assayed in this study included 51 isolates of *A. baumannii*, 27 of *Enterobacter cloacae*, 77 of *E. coli*, 59 of *K. pneumoniae* and 136 of *P. aeruginosa* obtained from individual patients in medical settings in Myanmar. These 350 isolates were obtained from 2016 to 2018 in 10 hospitals in Myanmar. Drug-susceptibilities of imipenem and meropenem were tested using the microdilution method according to the criteria of the Clinical Laboratory Standards Institute (CLSI) criteria [10]. Of a total of 350 isolates, 302 isolates (115 isolates of *P. aeruginosa*, 60 isolates of *E. coli*, 52 isolates of *K. pneumoniae*, 49 isolates of *A. baumannii*, and 26 isolates of *E. cloacae*) were resistant to carbapenems, including imipenem and/or meropenem. *E. coli* BL21-CodonPlus (DE3)-RIP (Agilent Technologies, Santa Clara, CA) were used for recombinant NDM-1, − 3, − 4, − 5, − 7, − 8, − 12 and − 13 proteins. Recombinant NDM-1 was prepared for rat anti-NDM-1 monoclonal antibodies (mAbs), and the other NDMs were prepared for evaluating whether the newly developed immunochromatographic kit can detect NDM variants.

### Genotyping of *bla*_NDMs_

The *bla*_NDM_ genes were amplified using PCR primers NDM-F (5′-ATGGAATTGCCCAATATTATG-3′) and NDM-R (5’TCAGCGCAGCTTGTCGGCCAT-3′). All PCR products were sequenced using an ABI 3500XL Genetic Analyzer (Applied Biosystems, Foster City, CA, USA). Other MBLs-encoding genes, including *bla*_VIMs_, *bla*_DIM-1_ and *bla*_IMPs_, were screened using PCR and sequencing as described previously [11, 12].

### Recombinant NDMs

The open reading frames of NDMs-1, − 3, − 4, − 5, − 7, − 8, − 12 and − 13, without the signal peptide region, were cloned into the pET28a expression vector (Novagen, Inc., Madison, WI, USA) using the primer set *Bam*HI-TEV-NDM-F (5′-ATGGATCCGAAAACCTGTATTTCCAAGGCCAGCAAATGGAAACTGGCGAC-3′) and *Xho*I-NDM-R (5′-ATCTCGAGTCAGCGCAGCTTGTCGGCCATG-3′). The resulting plasmids were used to transform *E. coli* BL21-CodonPlus (DE3)-RIP (Agilent Technologies, Santa Clara, CA, USA). Recombinant NDM-1 was purified simultaneously using Ni-NTA Agarose, according to the manufacturer’s instruction (Qiagen, Hilden, Germany). His-tags were removed by digestion with TurboTEV protease (Accelagen, San Diego, CA, USA), and untagged proteins were purified by an additional passage over Ni-NTA agarose. The purity of NDMs, which was estimated by SDS-PAGE, was greater than 90%. During the purification procedure, the presence of β-lactamase activity was monitored using nitrocefin (Oxoid Ltd., Basingstoke, UK).

### Preparation of monoclonal antibodies

Rat anti-NDM-1 monoclonal antibodies were prepared as described previously.^7^ Five 8-week-old female Wister rats were purchased from Oriental Yeast (Tokyo, Japan) and immunized with NDM-1. The immunized rats were enthanized using sodium pentobarbital injected intraperitoneally (200 mg/kg). Hybridomas were screened by enzyme-linked immunosorbent assays (ELISA).

### Sensitivity of the immunochromatographic assay

One hundred μL aliquots of serial 2-fold dilutions of overnight cultures of bacteria were mixed with 350 μL of alkaline solution containing 360 mM sodium hydroxide, 290 mM guanidine hydrochloride), supplemented with non-ionic detergents. After neutralization with acidic solution, 100 μL of the bacterial lysates were tested by the immunochromatographic assay. The number of colony-forming units (cfu) was determined by spreading aliquots of bacterial lysates onto blood agar plates.

### Assembly of the assay

The assay strips were prepared by laminating a nitrocellulose membrane, a colloidal gold-conjugated glass fiber, and an absorbent paper onto a polystyrene self-adhesive floor. To prepare the test lines, nitrocellulose membranes were coated with 0.35 μg of rat mAbs per test at a position 26 mm from the sample application area. To prepare the reference lines, the membranes were coated with 0.53 μg of anti-rodent IgG (Vector Laboratories, Burlingame, CA) per test at a position 32.5 mm from the sample application area. Colloidal gold-conjugated glass fibers were prepared by soaking glass filters in rat mAb conjugated to colloidal gold. The assay strips were stored in waterproof bags with a desiccant at room temperature until use.

Bacterial colonies grown on LB agar plates were picked with a swab and suspended in soft test tubes containing alkaline solution (360 mM sodium hydroxide, 290 mM guanidine hydrochloride) supplemented with non-ionic detergent, including polyoxyethylene (20) sorbitan monooleate. After neutralization, three drops (0.1 mL) of each bacterial lysate were added to the test plate, and the results analyzed by visual inspection 15 min later. The test plates with the results were photographed. The photos generated in this study are available from the corresponding author on reasonable request.

### Epitope mapping of mAbs

To determine putative epitopes of NDM-1 recognized by mAbs, short peptides consisting of 24–25-mers covering all amino acid sequences of NDM-1 without signal peptide (from aa 53 to aa 270) were synthesized (Table 1). The epitopes recognized by mAbs were determined as described [13].

**Table 1:** Immunochromatographic assay for detection of NDM-type MBLs in Gram-negative pathogens

Species	No. of isolates	Types of MBLs^a^	Immunochromotographic assay for detection of NDM-type MBLs
			Positive/isolates tested
*Acinetobacter baumannii*	5	NDM-1	5/5
	46	-^b^	0/46
*Pseudomonas* *aeruginosa*	23	NDM-1 (19/23), NDM-7 (4/23)	23/23
	24	IMP-1 (22/24), IMP-7 (2/24)	0/24
	19	VIM-1 (1/19), VIM-2 (16/19), VIM-5 (2/19)	0/19
	7	DIM-1	0/7
	63	-^b^	0/63
*Entereobacteriaceae*			
*Enterobacter* *cloacae*	25	NDM-1 (21/25), NDM-4 (2/25), NDM-5 (1/25), NDM-7 (1/25)	25/25
	2	-^b^	0/2
*Escherichia* *coli*	60	NDM-1 (6/60), NDM-4 (11/60), NDM-5 (39/60), NDM-7 (4/60)	60/60
	17	-^b^	0/17
*Klebsiella* *pneumoniae*	51	NDM-1 (29/51), NDM-4 (1/51), NDM-5 (15/51), NDM-7 (6/51)	51/51
	8	-^b^	0/8

^a^Types of MBLs were determined using PCR and sequencing as described previously [11, 12]

^b^MBLs non-producers

## Results

### Development of an immunochromatographic assay

**Fig. 1:**
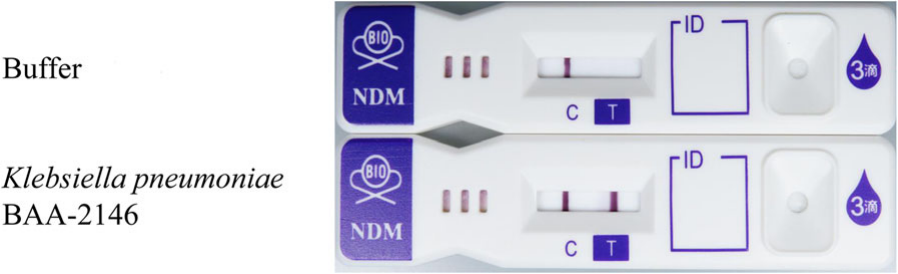
Immunochromatography using mAbs 1E2–7 and 4E4–4. In negative cases, a line appears only at the reference line (C). In positive cases, another line also appears at the positive test line (T) in addition to the reference line

We obtained 4 mAbs that reacted with recombinant NDM-1. These mAbs were used to design 12 immunochromatographic assay prototypes and their reactivities to NDM were evaluated (data not shown). The strongest intensity test was obtained with the assay consisting of mAb 1E2–7 immobilized on the membrane and mAb 4E4–4 labelled with colloidal gold. The immunochromatographic assay was therefore designed using these two mAbs (Fig. 1).

This assay detected 500 ng recombinant NDM-1 (500 ng) (data not shown), with a sensitivity of 250 ng recombinant NDM-1. To date, 21 variants of NDM-type MBL have been described (ftp://ftp.ncbi.nlm.nih.gov/pathogen/betalactamases/Allele.tab). Seven of these, NDM-3, − 4, − 5, − 7, − 8, − 12 and − 13, were positive on this assay (data not shown), indicating that the immunochromatographic assay detects several NDM variants.

### Identification of epitopes recognized by mAbs

Competition assays using two peptides, aa 37–148 and aa 147–270, covering the entire NDM-1 molecule, showed that both 1E2–7 and 4E4–4 bound to the aa 147–270 (Additional file 1: Figure S1). In addition, competition assays using six peptides, each 24 or 25 amino acids in length and covering the aa 147–270 region of NDM-1, found that both 1E2–7 and 4E4–4 bound to the aa 167–191 peptide, GWVEPATAPNFGPLKVFYPGPGHTS, which consists of β8 and β9 of NDM-1, indicating that both mAbs recognized β8 and/or β9 of NDM-1 (Additional file 2: Figure S2).

### Ability of the immunochromatographic assay to detect NDM producers in Myanmar

Testing showed that the NDM-1-producing strain *K. pneumoniae* BAA-2146 was positive (Fig. 1), with the sensitivity of this assay being 1.6 × 10^6^ cfu (data not shown).

A total of 350 Gram-negative pathogens, including 51 isolates of *Acinetobacter* species, 163 isolates of *Enterobacteriaceae* and 136 isolates of *Pseudomonas*, were tested using this immunochromatographic assay. Of the 350 isolates, 164 (46.9%) were positive on this assay (Table 1). The assay showed 100% specificity and 100% sensitivity (Table 1). These 164 isolates, including 5 of 51 *A. baumannii* isolates (9.8%), 25 of 27 *E. cloacae* isolates (92.6%), 60 of 77 *E. coli* isolates (77.9%), 51 of 59 *K. pneumoniae* isolates (86.4%) and 23 of 136 *P. aeruginosa* isolates (16.9%), harbored *bla*_NDMs_, whereas the remaining 186 isolates did not. The assay detected pathogens producing various NDM types, including NDM-1, − 4, − 5 and − 7, whereas it did not detected pathogens producing other MBLs, including DIM-1, IMP-1, IMP-7, VIM-1 and VIM-5 (Table 1). These results indicate that the immunochromatographic assay detects pathogens producing specific types of NDM-like MBLs.

Carbapenem-resistant *Enterobacteriaceae*, defined as having MICs ≥4 μg/ml to imipenem/meropenem, isolated in Myanmar harbored *bla*_NDMs_ with high probability of > 96%, i.e., 25 isolates of 26 carbapenem-resistant *E. cloacae* isolates (96.2%), 60 of 60 carbapenem-resistant *E. coli* isolates (100%), and 51 of 52 carbapenem-resistant *K. pneumoniae* isolates (98.1%) produced NDMs. The majority of *E. cloacae* and *K. pneumoniae* isolates in Myanmar produced NDM-1 (21/25 *E. cloacae* and 29/51 *K. pneumoniae*), whereas that of *E. coli* isolates produced NDM-5 (39/60). The proportions of NDM-1 producers in carbapenem-resistant *A. baumannii* and *P. aeruginosa*, defined as having MICs ≥8 μg/ml to imipenem/meropenem, were 10.2 and 20.0%, respectively (5/49 *A. baumannii* and 23/115 *P. aeruginosa*).

## Discussion

The immunochromatographic assay described in this study will likely be able to detect all NDM variants in clinical samples. The mAbs used in the assay recognized the β8 and/or β9 regions located on the surface of NDM-1 (Additional file 2: Figure S2), which include aa 189, a conserved residue at the active site of MBLs [14, 15]. Amino acid sequences surrounding the epitope from aa 155–232 are conserved in 20 of 21 NDM variants in the database (ftp://ftp.ncbi.nlm.nih.gov/pathogen/betalactamases/Allele.tab), all except NDM-18 [16]. Rather, NDM-18 had the amino acid substitution Glu170Lys in this region. Because NDM molecules evolve rapidly [14], it is unclear whether this assay will be able to detect future NDM variants. For example, although mAbs incorporated into an immunochromatographic assay to detect IMPs recognized two conserved regions and detected IMPs-1 to − 24 [17], IMP molecules evolved rapidly, with more than 60 variants developing since then (ftp://ftp.ncbi.nlm.nih.gov/pathogen/betalactamases/Allele.tab). Nevertheless, that immunochromatographic assay was able to detect all newly developed IMPs.

Our newly developed immunochromatographic detect NDM-production in various Gram-negative species including *Enterobacteriaceae* and glucose non-fermentative bacteria. In a previous study, a multiplex lateral flow immunoassay for the rapid identification of NDM-, KPC-, IMP- and VIM-type and OXA-48-like carbapenemase-producing *Enterobacteriaceae* was developed but *Pseudomonas* spp. or *Acinetobacter* spp. were not tested [18]. Because other studies describe emergence of NDMs in *Citrobacter* spp., *Morganella* spp., *Proteus* spp., *Providencia* spp., *Salmonella* spp., *Serratia marcescens*, *Shigella* spp., and *Vibrio* spp. [19–21], it is necessary to include these species in future studies.

Our present study indicates that NDMs-producing *Enterobacteriaceae*, including *E. cloacae*, *E. coli* and *K. pneumoniae*, disseminate in medical settings in Myanmar. In particular, NDM-5 was found in 39 of 60 carbapenem-resistant *E. coli* isolates. Homsey et al. reported that NDM-5 reduced the susceptibility of *E. coli* transformants to cephalosporins and carbapenems when compared with NDM-1 [22]. Moreover, it has been reported that clinical isolates of *E. coli* obtained in medical settings in Myanmar harbored *bla*_NDM-1_ in IncA/C_2_ plasmid, *bla*_NDM-4_ in IncX3 or IncFII, *bla*_NDM-5_ in IncFII and *bla*_NDM-7_ in IncX3, respectively [7].

It is necessary to perform the molecular epidemiological analysis in carbapenem-resistant Gram-negative bacteria harboring *bla*_NDMs_ genes obtained in medical settings in Myanmar. To collect and survey the NDM producing pathogens, the newly developed immunochromatographic assay for NDMs will be useful to detect NDM producers in clinical samples.

This assay can be detected NDM -producers within 15 min and can be used by both well- and poorly-equipped bacteriological laboratories. In future we will develop further immunochromatographic assays including other MBL genes (e.g. DIM, VIM) that are prevalent in Myanmar.

## Conclusion

The immunochromatographic assay is an easy-to-use and reliable kit for detection of NDMs-producing *Enterobacteriaceae* and glucose non-fermentative bacteria. The assay revealed that NDM-producing *Enterobacteriaceae*, including *E. cloacae*, *E. coli* and *K. pneumoniae*, isolates are wide-spread in medical settings in Myanmar.

## Additional files

Additional file 1: **Figure S1.** Determination of epitopes by ELISA. Competition assays using amino acids (aa) 37 to 148 and aa 147 to 270, covering the whole region of NDM-1, revealed that both 1E2–7 and 4E4–4 bound to the peptide from aa 147 to 270. When competition assays were conducted using 6 peptides with 24 or 25 amino acids, covering the region of NDM-1 from aa 147 to 270, both 1E2–7 and 4E4–4 bound to a peptide from aa 167 to 191. (TIF 526 kb)

Additional file 2 **Figure S2.** The mAbs used in the assay recognized the β8 and/or β9 regions (in red) located on the surface of NDM-1. (TIF 6352 kb)

## Data Availability

The datasets used and/or analyzed during the current study available from the corresponding author on reasonable request.

## Abbreviations

ELISA: Enzyme-linked immunosorbent assay
mAb: Monoclonal antibody
MBL: Metallo-β-lactamase
NDM: New Delhi metallo-β-lactamase
PCR: Polymerase chain reaction
SDS-PAGE: Sodium dodecyl sulfate-polyacrylamide gel electrophoresis
