# Supplementary figures and images for: Assessment of a newly developed immunochromatographic assay for NDM-type metallo-β-lactamase producing Gram-negative pathogens in Myanmar
Source: BMC Infect Dis. 2019 Jun 28;19:565. doi: 10.1186/s12879-019-4147-4 (PMC6599368; doi:10.1186/s12879-019-4147-4)

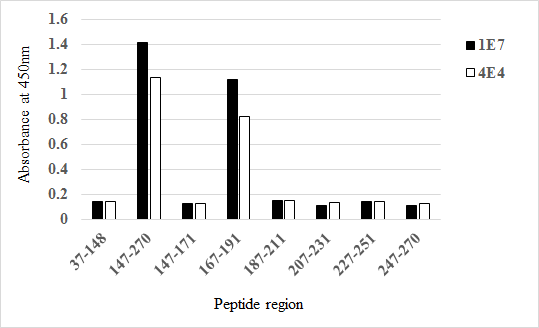

Supplement: Supplementary file 1 — Figure S1. Determination of epitopes by ELISA. Competition assays using amino acids (aa) 37 to 148 and aa 147 to 270, covering the whole region of NDM-1, revealed that both 1E2–7 and 4E4–4 bound to the peptide from aa 147 to 270. When competition assays were conducted using 6 peptides with 24 or 25 amino acids, covering the region of NDM-1 from aa 147 to 270, both 1E2–7 and 4E4–4 bound to a peptide from aa 167 to 191. (TIF 526 kb) [file 12879_2019_4147_MOESM1_ESM.tif]

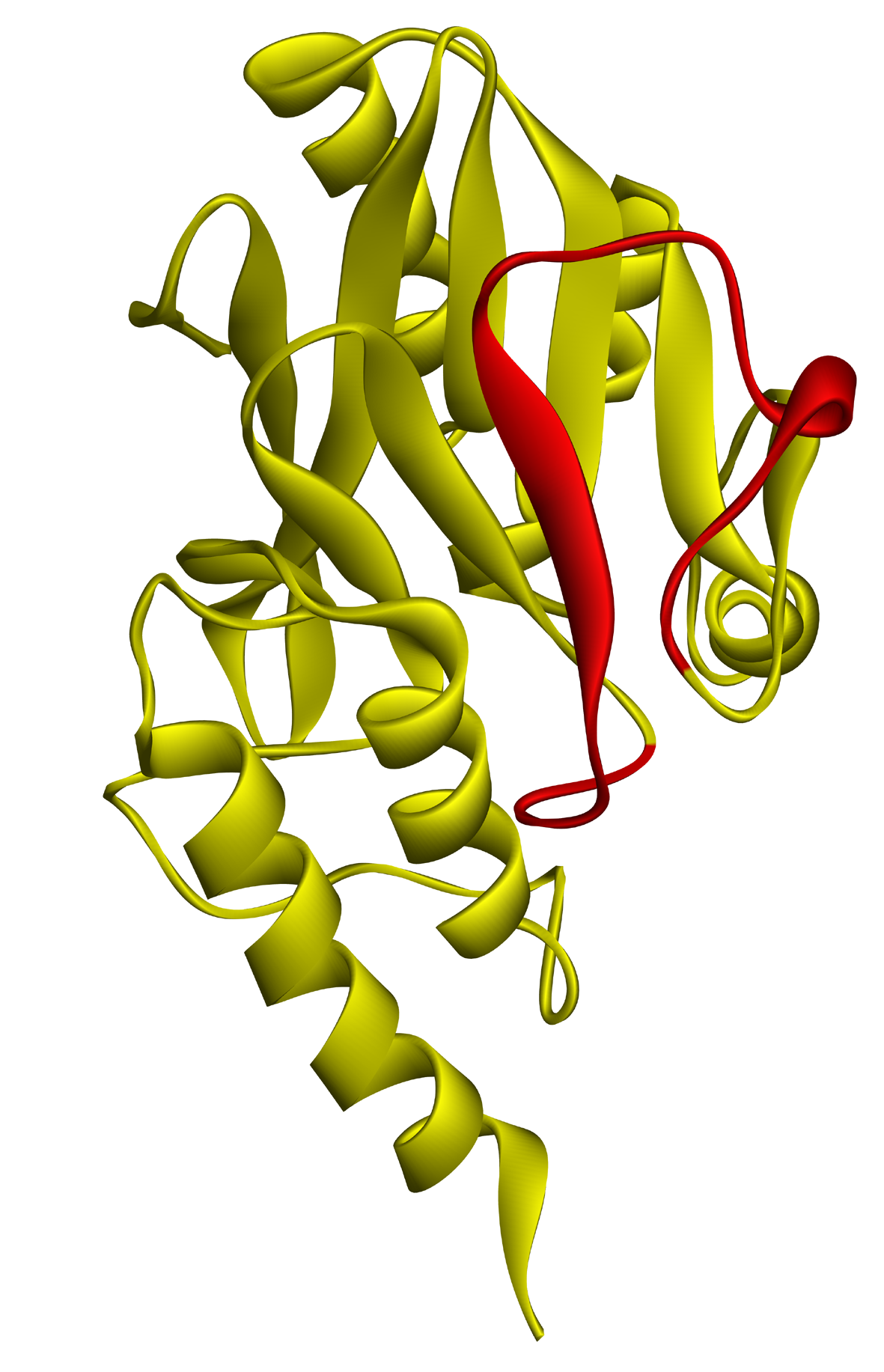

Supplement: Supplementary file 2 — Figure S2. The mAbs used in the assay recognized the β8 and/or β9 regions (in red) located on the surface of NDM-1. (TIF 6352 kb) [file 12879_2019_4147_MOESM2_ESM.tif]
